# Factors Influencing Physical Performance and Quality of Life in Post-COVID-19 Patients

**DOI:** 10.3390/diseases13040120

**Published:** 2025-04-19

**Authors:** Ajchamon Thammachai, Patchareeya Amput, Sirima Wongphon

**Affiliations:** 1 Department of Physical Therapy, School of Allied Health Sciences, University of Phayao, Phayao 56000, Thailand; ajchamon.th@up.ac.th; 2 Unit of Excellence of Human Performance and Rehabilitations, University of Phayao, Phayao 56000, Thailand; 3 Department of Traditional Chinese Medicine, School of Public Health, University of Phayao, Phayao 56000, Thailand; sirima.wo@up.ac.th

**Keywords:** post-COVID-19, quality of life, muscle strength, respiratory system, body mass index

## Abstract

**Background**: This study aims to identify the factors related to demographic variables and physical performance associated with quality of life (QoL) in post-COVID-19 pa-tients who have recovered from mild infection and were not hospitalized. **Methods**: Seventy-four post-COVID-19 individuals who recovered from mild COVID-19 infec-tion were assessed for the baseline demographic variables (age, sex, height, weight, body mass index; BMI) and clinical information (comorbidities, duration of COVID-19 infection, and exercise habits). Vital signs (heart rate, blood pressure, and oxygen sat-uration; SpO_2_) were measured. Physical performance was evaluated for upper- and lower-limb muscle strength, ability of balance, and cardiorespiratory performance. All participants were assessed for QoL. **Results**: Hand grip strength was negatively asso-ciated with gender and age while positively associated with the duration of COVID-19. Quadricep strength also showed a negative association with gender and duration of COVID-19. Age was positively associated with multiple quality of life dimensions, while emotional role limitations were negatively associated with the duration of COVID-19 and waist circumference. Mental health was negatively linked to BMI. **Conclusions**: This study highlights the complex impact of COVID-19 on physical per-formance and QoL, revealing that older adults often report better QoL despite reduced muscle strength, particularly in women. The findings emphasize the need for targeted rehabilitation programs addressing both physical and emotional health for vulnerable groups.

## 1. Introduction

Post-COVID-19 is defined as symptoms remaining longer than 12 weeks following the acute onset of COVID-19 [1]. The symptoms of post-COVID-19 could present in all ages of patients with mild-intensity COVID-19 and who have not had any history of chronic disease [2,3]. Mental and cognitive impairments, pain, fatigue, muscle weakness, disturbed sleep, and dyspnea are the most common symptoms found in post-COVID-19 patients [2,4,5]. A previous study reported that older adults with co-morbidities including diabetes, cancer, or cardiorespiratory disease and who were admitted to hospital during COVID-19 infection were more likely to develop serious clinical outcomes after COVID-19 infection [6]. The cause of symptoms of post-COVID-19 patients is due to severe acute respiratory syndrome coronavirus 2 (SARS-CoV-2) virus persistence and immune dysregulation, resulting in long-term tissue damage and inflammation [7]. Moreover, several studies have reported that post-COVID-19 patients express decreased mobility, impaired ability to carry out daily activities, discomfort or pain, depression, and anxiety at four months to one-year follow-up after hospitalization [8,9,10]. These impairments lead to reduced quality of life (QoL) in these populations [10]. Additionally, comorbidity, body mass index (BMI), and education factors were associated with QoL in post-COVID-19 patients who required hospitalization [11]. However, this evidence has few reports in patients with mild COVID-19 infection and patients who had no hospital admission.

Information on factors influencing physical performance and QoL in patients who have recovered from mild COVID-19 infection and those who were not hospitalized is interesting. This information helps us to understand the factors that impact QoL in these patients. In addition, previous studies have focused on the pathophysiology of the COVID-19 disease and the characteristic persistent symptoms in this population [12]. Furthermore, a previous study found that older adults recovering from mild COVID-19 decreased their balance ability using the timed up-and-go (TUG) test and QoL using the Short Form-36 (SF-36) when compared to older adults without a history of COVID-19 [13]. The decreased balance ability in these patients, due to SARS-CoV-2-activated neuroinflammation from the influx of cytokines into different sites of the central nervous system (CNS), resulted in postural balance impairment [14]. Moreover, the reduced QoL in these patients may be due to their decreased balance ability. However, their study did not evaluate factors that can influence QoL in these patients [13]. Therefore, this study aims to identify the demographic variables and physical performance factors associated with QoL in post-COVID-19 patients who have recovered from mild infections and were not hospitalized.

## 2. Materials and Methods

### 2.1. Study Design

A cross-sectional study design was used to identify the factors of demographic variables and physical performance associated with QoL in post-COVID-19 patients who have recovered from mild infections and were not hospitalized.

### 2.2. Participants

Seventy-four post-COVID-19 individuals with recovery from mild COVID-19 infection were used in the study. The sample size was calculated using a power of 0.80, power analysis with an alpha of 0.05, and an effect size dz of 0.30 [15]. The participants who were recruited were aged 18 years or above with post-COVID-19 and had confirmed SARS-CoV-2 infection lasting longer than 12 weeks using the polymerase chain reaction (PCR) or antigen test kit (ATK) before the investigation procedure. Participants with musculoskeletal, cardiorespiratory, or neurological diseases that could affect their test performance were excluded from the study. All participants provided written informed consent after receiving detailed information about the study’s objectives and methods. This study was approved by the Clinical Research Ethics Committee of the University of Phayao, Phayao, Thailand (HREC-UP-HSST 1.2/110/67), with approval granted on 16 July 2024.

### 2.3. Procedure

All participants underwent baseline assessments, including demographic variables (age, sex, height, weight, body mass index; BMI) and clinical information (comorbidities, duration of COVID-19 infection, and exercise habits). Vital signs (heart rate, blood pressure, and oxygen saturation; SpO_2_) were measured. Physical performance evaluation included assessments of upper- and lower-limb muscle strength, balance ability, and cardiorespiratory performance. All participants were assessed for QoL. Test sequences were randomly assigned using the website randomizer.org.

#### 2.3.1. Hand Grip Test

Each participant stood with their elbow extended during the test. They performed three consecutive grip strength trails, each lasting 3 s, with 15 s of rest period between repetitions. The highest peak force recorded was used for analysis [16].

#### 2.3.2. Quadriceps Muscle Strength Test

Quadriceps muscle strength was measured using a hand-held dynamometer (Model-01165, Lafayette Instrument Company, Lafayette, IN, USA). Participants sat with their hip and knee flexed at 90 degrees, and maximal isometric quadriceps strength (in kilograms) was assessed in the dominant leg during knee extension. Each measurement was repeated at least three times, and the highest recorded value was documented [17].

#### 2.3.3. Six-Min Walk Test (6MWT)

Participants were instructed to walk at a self-selected pace for six minutes, covering as much distance as possible in an uninterrupted 30 m corridor. They were allowed to stop and rest if needed before resuming the test. Verbal encouragement was provided throughout the assessment [18].

#### 2.3.4. Timed Up-and-Go Test (TUG)

Participants were instructed to rise from a chair upon signal, walk to a designated marker, go around it, return to the chair, and sit down as quickly as possible. They began the test in a seated position with an upright posture, hands resting on their thighs, and feet flat on the ground. Participants were reminded that the test was timed and that they should walk as quickly as possible without running [19].

#### 2.3.5. Quality of Life (QoL)

QoL was evaluated using the Short Form-36 (SF-36) questionnaire, which assesses eight dimensions: physical functioning, physical role limitations, bodily pain, general health perceptions, vitality, social functioning, emotional role limitations, and mental health. The total score ranges from 0 to 100, with higher scores indicating better QoL [20].

### 2.4. Statistical Analysis

Descriptive statistics were used to present demographic data. A non-parametric test does not assume anything about the underlying distribution. Multiple linear regression analysis was used to examine factors associated with post-COVID-19 physical performance and quality of life among patients. Potential covariates (univariate analysis, *p* < 0.1) were included in the multiple regression model. Covariates for physical performance included gender, age, duration of COVID-19, exercise days, waist circumference, hip circumference, and BMI. Covariates for quality of life after COVID-19 included health status, age, duration of COVID-19, exercise days, waist circumference, hip circumference, and BMI. Inferential statistics were represented by beta (*β*), standard error (SE), and a 95% confidence interval (95% CI). The level of significance was set at *p* < 0.05.

## 3. Results

The study involved 74 participants, 68.90% female and 31.10% male, with 83.80% reporting no underlying diseases. The average age was 43.11 years, and the mean BMI was 21.38 kg/m^2^. The average waist circumference was 70.01 cm, and hip circumference was 89.86 cm. Vital signs were within normal ranges, and participants exercised, on average, 1.51 days per week. The results are detailed in Table 1.

Table 2 presents the factors associated with physical performance following COVID-19 recovery in the sample of 74 participants. The multivariate regression analysis revealed that hand grip strength was negatively associated with both gender and age, while showing a positive association with the duration of COVID-19. Specifically, the coefficients were *β* ± SE = −3.33 ± 0.75 (95% CI = −4.82, −1.83) for gender, *β* ± SE = −0.05 ± 0.02 (95% CI = −0.09, −0.02) for age, and *β* ± SE = 0.18 ± 0.09 (95% CI = 0.01, 0.35) for COVID-19 duration. For quadriceps strength, significant negative associations were found with gender and COVID-19 duration, with coefficients of *β* ± SE = −5.38 ± 0.63 (95% CI = −6.65, −4.12) for gender and *β* ± SE = −0.15 ± 0.07 (95% CI = −0.25, −0.01) for COVID-19 duration. Additionally, both the TUG test and the distance covered in the 6MWT were negatively associated with age, with coefficients of *β* ± SE = 0.09 ± 0.01 (95% CI = 0.07, 0.10) for TUG and *β* ± SE = −2.24 ± 0.27 (95% CI = −2.78, −1.71) for the 6MWT distance.

Table 3 summarizes the factors associated with QoL following COVID-19 recovery in a sample of 74 participants. The multivariate regression analysis indicated that age was positively associated with several QoL dimensions, including physical function (*β* ± SE = 0.18 ± 0.51; 95% CI = 0.08, 0.28), physical role limitations (*β* ± SE = 0.18 ± 0.05; 95% CI = 0.86, 0.26), bodily pain (*β* ± SE = 0.19 ± 0.05; 95% CI = 0.09, 0.28), general health perception (*β* ± SE = 0.19 ± 0.04; 95% CI = 0.11, 0.27), vitality (*β* ± SE = 0.15 ± 0.04; 95% CI = 0.07, 0.24), social functioning (*β* ± SE = 0.24 ± 0.03; 95% CI = 0.17, 0.31), emotional role limitations (*β* ± SE = 0.21 ± 0.02; 95% CI = 0.16, 0.25), and mental health (β ± SE = 0.14 ± 0.03; 95% CI = 0.08, 0.20). Conversely, emotional role limitations showed negative associations with both COVID-19 duration (*β* ± SE = −0.26 ± 0.10; 95% CI = −0.46, −0.07) and waist circumference (*β* ± SE = −0.80 ± 0.32; 95% CI = −1.44, −0.17). Additionally, mental health was negatively associated with BMI (*β* ± SE = −0.41 ± 0.15; 95% CI = −0.70, −0.12).

## 4. Discussion

This study explored the association between various factors and physical performance, as well as QoL, in post-COVID-19 patients who had recovered from mild infections and were not hospitalized. The 74 post-COVID-19 patients had an average age of 43.11 years, with key baseline characteristics such as a mean BMI of 21.38 kg/m^2^ and an average waist circumference of 70.01 cm. Participants exercised an average of 1.51 days per week, underscoring the key factors connected to physical rehabilitation and QoL outcomes after COVID-19.

The findings of this study provide insights into the factors influencing physical performance in individuals recovering from COVID-19. The multivariate regression analysis identified significant associations between gender, age, duration of COVID-19, and various aspects of physical performance. Our results found that the hand-grip strength test was negatively correlated with both gender and age, indicating that women and older adults exhibited lower- and upper-limb muscle strength. This observation is consistent with prior research showing that women generally have lower hand grip strength compared to men, and that grip strength declines with age due to natural muscle mass loss [21,22]. Interestingly, the duration of COVID-19 showed a positive association with hand grip strength. This may suggest that individuals who experienced COVID-19 for a longer duration might have had more opportunities for recovery and muscle adaptation, potentially as a result of engaging in rehabilitation activities or increased awareness of their health status during a prolonged recovery period [23,24]. In contrast, quadriceps muscle strength showed significant negative associations with both gender and the duration of COVID-19. Women and those experiencing a longer duration of COVID-19 showed diminished quadriceps muscle strength. This may be attributed to the prolonged effects of COVID-19 on muscle function, as muscle atrophy and weakness are prevalent in extended illness, particularly for those with extended recovery periods [23,25]. The greater reduction in quadriceps muscle strength compared to hand grip strength may reflect the impact of inactivity on larger muscle groups in the legs during the illness [23]. Furthermore, both the TUG test and the 6MWT were negatively associated with age, indicating that older participants had slower TUG times and covered shorter distances in the 6MWT. This is consistent with age-related declines in mobility, balance, and endurance [19,26], which may have been exacerbated by the deconditioning effects of COVID-19 [27]. Older adults may experience more difficulty in regaining their pre-illness levels of physical performance due to factors like muscle loss, joint stiffness, and a more prolonged recovery process [28]. The negative associations between age, gender, and quadriceps strength, combined with the reductions in mobility observed in the TUG and 6MWT, underscore the importance of targeted interventions. Older adults and women, in particular, may be at a higher risk of reduced physical performance following COVID-19 recovery. Tailored rehabilitation programs that focus on rebuilding muscle strength and improving mobility are vital in helping these individuals regain functional independence. These findings emphasize the need for long-term physical rehabilitation strategies post-COVID-19, especially for vulnerable groups such as older adults and women, who may face greater challenges in recovering their physical performance. Additionally, the role of early rehabilitation and consistent physical activity during recovery should be further explored to mitigate long-term impacts and support a quicker return to pre-COVID-19 functioning. Incorporating pacing techniques into rehabilitation programs can help prevent symptom exacerbation and facilitate a more sustainable recovery process, ensuring that patients gradually regain their strength without overexertion.

The results of this study provide valuable insights into the factors affecting QoL in individuals recovering from COVID-19. Multivariate regression analysis revealed that age was positively associated with several dimensions of QoL, such as physical function, physical role limitations, bodily pain, general health perception, vitality, social functioning, emotional role limitations, and mental health. These findings suggest that older adults reported better QoL in multiple areas, which could be attributed to their resilience [29,30] and coping mechanisms, as well as potentially lower expectations of physical health compared to younger individuals [31,32]. Therefore, this contrasts with the general expectation that older age may be linked to lower QoL, highlighting the complexity of post-COVID-19 recovery. Emotional role limitations were negatively associated with both the duration of COVID-19 and waist circumference. This suggests that prolonged illness may have affected individuals’ emotional well-being, limiting their capacity to fulfill social or emotional roles [33]. A larger waist circumference, associated with central obesity, has also been linked to poorer emotional health and self-esteem, explaining this negative association [34,35]. Additionally, mental health was negatively associated with BMI, indicating that higher BMI levels might correlate with poorer mental health outcomes after COVID-19 recovery. This finding aligns with previous research suggesting that obesity is a risk factor for both physical and psychological health issues, particularly given the adverse effects of prolonged illness, obesity, and central adiposity on QoL [36,37]. Therefore, these results underscore the need to address both physical and emotional health in post-COVID-19 recovery. These findings highlight the importance of comprehensive rehabilitation programs that focus on physical recovery while also addressing emotional and mental health concerns, especially for individuals who had longer COVID-19 illness durations or higher BMI and waist circumference.

Our study may be limited by its sample size, as certain variables could potentially achieve statistical significance with a larger and more appropriate sample.

## 5. Conclusions

This study demonstrates the complex effects of COVID-19 on physical performance and QoL in individuals recovering from mild infections. It finds that older adults often report better QoL, possibly due to resilience and coping mechanisms. However, women and older adults exhibited reduced muscle strength, particularly in hand grip and quadriceps. These findings underscore the necessity for targeted rehabilitation programs that address both physical and emotional health, especially for vulnerable populations like older adults and women.

## Figures and Tables

**Table 1:** Demographic data of participants (*n =* 74).

Parameters		*n* (%)	Mean ± SD (Min–Max)
**Gender**	**Male** **Female**	23 (31.10) 51 (68.90)	
**Health status**	**Underlying disease** **No underlying diseases**	12 (16.20) 62 (83.80)	
**Age**			43.11 ± 17.23 (20.00–65.00)
**Weight** (**kg**)			57.07 ± 9.72 (6.00–18.00)
**Height** (**m**)			1.63 ± 0.07 (1.50–1.78)
**Body mass index**			21.38 ± 2.38 (16.73–25.95)
**Waist circumference** (**cm**)			70.01 ± 4.11 (60.00–78.00)
**Hip circumference** (**cm**)			89.86 ± 5.31 (78.00–99.00)
**Systolic blood pressure** (**mmHg**)			128.26 ± 10.43 (101.0–141.0)
**Diastolic blood pressure** (**mmHg**)			77.11 ± 7.26 (61.0–98.0)
**Heart rate** (**bpm**)			82.12 ± 8.62 (61.0–97.0)
**O_2_ saturation** (**%**)			98.35 ± 0.65 (97.0–99.0)
**Frequency of exercise** (**day**)			1.51 ± 1.09 (0.00–3.00)

Denote: *n* = number; F = female; M = male; kg = kilograms; m = meters; cm = centimeters.

**Table 2:** Factors associated with physical performance after COVID-19 recovery (*n* = 74).

	*β ± SE (95% CI)*			
*Factors*	*Hand Grip* *(kg)*	*Quadriceps Strength* *(kg)*	*TUG*	*Distance* *6MWT*
**Gender**	*−3.33 ± 0.75*	*−5.38 ± 0.63*	*−0.33 ± 0.31*	*22.48 ± 11.34*
	(−4.82, −1.83) **	(−6.65, −4.12) **	*(−0.95, 0.28)*	*(−0.16, 45.12)*
***p-value***	<0.001 **	<0.001 **	*0.283*	*0.052*
**Age** (**year**)	*−0.05 ± 0.02*	*−0.02 ± 0.02*	*0.09 ± 0.01*	*−2.24 ± 0.27*
	(−0.09, −0.02) **	*(−0.05, 0.01)*	(0.07, 0.10) **	*(* *−* 2.78, −1.71) **
***p-value***	0.006 *	*0.145*	<0.001 **	*0.545*
**Duration of COVID-19** (**month**)	*0.18 ± 0.09*	*−0.15 ± 0.07*	*0.02 ± 0.04*	*−0.79 ± 1.29*
	(0.01, 0.35) *	(−0.25, −0.01) *	*(−0.05, 0.09)*	*(−3.38, 1.80)*
***p-value***	0.036 *	0.044 *	*0.552*	*0.545*
**Exercise days** (**day**)	*−0.07 ± 0.27*	*0.21 ± 0.23*	*0.15 ± 0.11*	*−2.92 ± 4.03*
	*(−0.60, 0.46)*	*(−0.24, 0.65)*	*(−0.06, 0.37)*	*(−10.96, 5.13)*
***p-value***	*0.794*	*0.362*	*0.168*	*0.472*
**Waist circumference** (**cm**)	*−0.12 ± 0.10*	*−0.03 ± 0.09*	*−0.02 ± 0.04*	*−2.31 ± 1.57*
	*(−0.33, 0.09)*	*(−0.21, 0.14)*	*(−0.11, 0.07)*	*(−5.05, 0.82)*
***p-value***	*0.253*	*0.715*	*0.632*	*0.146*
**Hip circumference** (**cm**)	*−0.05 ± 0.08*	*−0.05 ± 0.06*	*0.05 ± 0.03*	*−0.04 ± 1.15*
	*(−0.20, 0.10)*	*(−0.17, 0.08)*	*(−0.01, 0.12)*	*(−2.33, 2.24)*
***p-value***	*0.513*	*0.463*	*0.097*	*0.971*
**Body mass index** (**BMI**)	*−0.07 ± 0.13*	*0.03 ± 0.11*	*−0.04 ± 0.06*	*1.40 ± 2.02*
	*(−0.34, 0.19)*	*(−0.19, 0.26)*	*(−0.15, 0.07)*	*(−2.63, 5.42)*
***p-value***	*0.591*	*0.765*	*0.484*	*0.490*

Values are presented as unstandardized beta (*B*) and standard error (SE), confidence interval (95%). * p < 0.05, ** p < 0.001.

**Table 3:** Factors associated with quality of life after COVID-19 recovery (*n* = 74).

*Factors*	*β ± SE (95% CI)*							
	Physical Function	Physical Role Limitations	Bodily Pain	General Health Perceptions	Vitality	Social Functioning	Emotional Role Limitation	Mental Health
**Health status**	*1.80 ± 1.98*	*−1.82 ± 1.73*	*−3.02 ± 1.81*	*−0.25 ± 1.48*	*−1.36 ± 1.70*	*0.40 ± 1.31*	*1.73 ± 0.95*	*0.67 ± 1.08*
	*(−2.15, 5.76)*	*(−5.27, 1.63)*	*(−6.62, 0.59)*	*(−3.22, 2.71)*	*(−4.76, 2.04)*	*(−2.21, 3.02)*	*(−1.16, 3.63)*	*(−1.50, 2.83)*
***p-value***	*0.366*	*0.296*	*0.100*	*0.866*	*0.427*	*0.759*	*0.073*	*0.541*
**Age (year)**	*0.18 ± 0.51*	*0.18 ± 0.05*	*0.19 ± 0.05*	*0.19 ± 0.04*	*0.15 ± 0.04*	*0.24 ± 0.03*	*0.21 ± 0.02*	*0.14 ± 0.03*
	(0.08, 0.28) *	(0.86, 0.26) *	(0.09, 0.28) **	(0.11, 0.27) **	(0.07, 0.24) *	(0.17, 0.31) **	(0.16, 0.25) **	(0.08, 0.20) **
***p-value***	0.001 *	<0.001 **	<0.001 **	<0.001 **	<0.001 **	<0.001 **	<0.001 **	<0.001 **
**Duration of COVID-19 (month)**	*−0.02 ± 0.21*	*0.11 ± 0.18*	*−0.03 ± 0.19*	*0.04 ± 0.15*	*0.23 ± 0.18*	*0.32 ± 0.14*	*−0.26 ± 0.10*	*0.08 ± 0.11*
	*(−0.43, 0.39)*	*(−0.25, 0.47)*	*(−0.41, 0.35)*	*(−0.27, 0.35)*	*(−0.12, 0.59)*	(0.04, 0.59) *	(−0.46, −0.07) *	*(−0.15, 0.31)*
***p-value***	*0.928*	*0.538*	*0.883*	*0.784*	*0.195*	0.023 *	0.010 *	*0.478*
**Exercise days (day)**	*−0.51 ± 0.67*	*−0.41 ± 0.58*	*−0.01 ± 0.61*	*−0.63 ± 0.50*	*1.15 ± 0.57*	*−0.05 ± 0.44*	*−0.80 ± 0.32*	*0.65 ± 0.36*
	*(−1.84, 0.82)*	*(−1.57, 0.75)*	*(−1.22, 1.20)*	*(−1.63, 0.36)*	*(0.01, 2.29)*	*(−0.93, 0.83)*	(−1.44, −0.17) *	*(−0.08, 1.38)*
***p-value***	*0.488*	*0.485*	*0.988*	*0.209*	0.048 *	*0.915*	0.014 *	*0.079*
**Waist circumference (cm)**	*0.04 ± 0.25*	*0.12 ± 0.22*	*0.17 ± 0.23*	*0.20 ± 0.19*	*−0.21 ± 0.22*	*0.01 ± 0.17*	*0.04 ± 0.12*	*−0.27 ± 0.13*
	*(−0.47, 0.53)*	*(−0.32, 0.56)*	*(−0.29, 0.63)*	*(−0.18, 0.58)*	*(−0.64, 0.22)*	*(−0.32, 0.34)*	*(−0.20, 0.28)*	*(−0.54, 0.01)*
***p-value***	*0.886*	*0.593*	*0.455*	*0.289*	*0.332*	*0.946*	*0.750*	*0.054*
**Hip** **circumference (cm)**	*−0.10 ± 0.17*	*−0.10 ± 0.15*	*0.08 ± 0.16*	*−0.05 ± 0.13*	*0.06 ± 0.15*	*−0.10 ± 0.12*	*−0.15 ± 0.08*	*0.15 ± 0.10*
	*(−0.44, 0.25)*	*(−0.39, 0.21)*	*(−0.24, 0.39)*	*(−0.31, 0.21)*	*(−0.23, 0.36)*	*(−0.32, 0.14)*	*(−0.31, 0.02)*	*(−0.45, 0.33)*
***p-value***	*0.582*	*0.533*	*0.619*	*0.696*	*0.670*	*0.411*	*0.084*	*0.132*
**Body mass index (BMI)**	*−0.42 ± 0.27*	*0.22 ± 0.23*	*0.13 ± 0.24*	*0.39 ± 0.20*	*0.28 ± 0.23*	*0.13 ± 0.18*	*−0.08 ± 0.13*	*−0.41 ± 0.15*
	*(−0.95, 0.11)*	*(−0.25, 0.68)*	*(−0.36, 0.62)*	*(−0.01, 0.78)*	*(−0.18, 0.73)*	*(−0.22, 0.48)*	*(−0.33, 0.18)*	(−0.70, −0.12) *
***p-value***	*0.116*	*0.355*	*0.587*	*0.056*	*0.230*	*0.473*	*0.554*	0.006 *

Values are presented as unstandardized beta (*B*) and standard error (SE), confidence interval (95%). * p < 0.05, ** p < 0.001.

## Data Availability

Data are contained within the article.
